# The DNA damage response network in the treatment of head and neck squamous cell carcinoma

**DOI:** 10.1016/j.esmoop.2021.100075

**Published:** 2021-03-10

**Authors:** A. Psyrri, M. Gkotzamanidou, G. Papaxoinis, L. Krikoni, P. Economopoulou, I. Kotsantis, M. Anastasiou, V.L. Souliotis

**Affiliations:** 1 Section of Medical Oncology, Department of Internal Medicine, Faculty of Medicine, National and Kapodistrian University of Athens, Attikon University Hospital, Athens, Greece; 2 Agios Savvas Anticancer Hospital, Athens, Greece; 3 Institute of Chemical Biology, National Hellenic Research Foundation, Athens, Greece; 4 First Department of Propaedeutic Internal Medicine and Joint Rheumatology Program, National and Kapodistrian University of Athens, Athens, Greece

**Keywords:** DNA damage response, endogenous DNA damage, oxidative stress, head and neck squamous cell carcinoma, cisplatin-containing chemoradiation, olaparib-containing regimens

## Abstract

**Background:**

We sought to determine whether DNA damage response (DDR)-related aberrations predict therapeutic benefit in cisplatin-treated head and neck squamous cell carcinoma (HNSCC) patients and how DDR pathways are modulated after treatment with olaparib alone or in combination with cisplatin or durvalumab.

**Patients and methods:**

Oxidative stress, abasic sites and DDR-related parameters, including endogenous DNA damage, DNA repair mechanisms and apoptosis rates, were evaluated in HNSCC cell lines and peripheral blood mononuclear cells from 46 healthy controls (HC) and 70 HNSCC patients at baseline and following treatment with cisplatin-containing chemoradiation or nivolumab or enrolled in the OPHELIA phase II trial (NCT02882308; olaparib alone, olaparib plus cisplatin, olaparib plus durvalumab).

**Results:**

HNSCC patients at diagnosis exhibited deregulated DDR-related parameters and higher levels of oxidative stress and abasic sites compared with HC (all *P* < 0.05). Accordingly, nucleotide excision repair (NER; *ERCC1, ERCC2/XPD, XPA, XPC*) and base excision repair (*APEX1, XRCC1*) genes were downregulated in patients versus HC whereas double-strand breaks repair (*MRE11A, RAD50, RAD51, XRCC2*) and mismatch repair (*MLH1, MSH2, MSH3*) genes were overexpressed. Corresponding results were obtained in cell lines (all *P* < 0.001). Excellent correlations were observed between individual *ex vivo* and *in vivo*/therapeutic results, with cisplatin non-responders showing higher levels of endogenous DNA damage, augmented oxidative stress and abasic sites, increased NER capacities and reduced apoptosis than responders (all *P* < 0.05). Also, longer progression-free survival correlated with lower NER capacity (*P* = 0.037) and increased apoptosis (*P* = 0.029). Interestingly, treatment with olaparib-containing regimens results in the accumulation of cytotoxic DNA damage and exerts an extra antitumor effect by elevating oxidative stress (all *P* < 0.05). Nivolumab induced no significant changes in the DDR parameters examined.

**Conclusions:**

Aberrations in DDR signals are implicated in the response to HNSCC chemotherapy and can be exploited as novel therapeutic targets, sensitive/effective non-invasive biomarkers as well as for the design of novel clinical trials.

## Highlights

• HNSCC patients at diagnosis show defective DDR network and higher levels of oxidative stress compared with HC.
• DDR aberrations and oxidative stress status in PBMCs appear to correlate with the response to cisplatin-based therapy.
• Olaparib treatment modulates DDR network and exerts extra antitumor effect by elevating oxidative stress in HNSCC patients.
• DDR aberrations in PBMCs may potentially be exploited as novel therapeutic targets and non-invasive predictive biomarkers.

## Introduction

Head and neck squamous cell carcinoma (HNSCC), the sixth most common cancer worldwide, is a heterogeneous disease that includes cancers involving the oral cavity, pharynx and larynx. HNSCC incidence is affected by age, genetic factors, geographic region and different lifestyle factors, including alcohol, smoking, betel quid use, oral hygiene and human papillomavirus (HPV) infection.^1^ HNSCC is considered one of the malignancies with the most severe impact on the quality of life of patients, caused mainly by the severe side-effects of treatment.^2^ Although HNSCC diagnosis and treatment have greatly improved with treatment advances, patient prognosis and quality of life remain poor.

The human genome is constantly subjected to endogenous and exogenous sources of damage.^3^ Exogenous sources of DNA damage include environmental agents such as ultraviolet light, ionizing radiation, chemicals, toxins and pollutants, while major sources of endogenous DNA damage include reactive oxygen species (ROS), aldehydes derived from lipid peroxidation, methylating agents, hydrolytic deamination and carbonyl stress.^4^ Endogenous damage may also arise due to genotoxic stress from cellular processes such as transcription and replication processes that overwhelm the high-fidelity of DNA repair in an otherwise repair-competent background.^3^

Protection against these genotoxic insults is secured by the network of DNA damage response (DDR) pathways triggered by the detection of DNA lesions.^5^ The subsequent step is the initiation of a signal transduction cascade including molecules that activate genome-protection pathways, such as DNA repair, cell cycle control, apoptosis, transcription and chromatin remodeling. Failure to repair DNA damage can result in a variety of genomic alterations, such as point mutations, chromosomal translocations and gain or loss of chromosomal segments or entire chromosomes.^6^ Under certain conditions, these genomic aberrations induce changes in cellular physiology that drive tumor initiation.^7^ In addition to playing a substantial role in tumor initiation, loss of DNA repair fidelity has important implications for tumor evolution and response to treatment. Common tumor characteristics, including high levels of oxidative stress, replicative stress and loss of DNA damage-induced cell cycle checkpoints, contribute to DNA damage accumulation.^8^^,^^9^ Besides high levels of endogenous DNA damage, functional loss of one or more DNA repair pathways is frequent in tumors.^10^ Due to the common combination of increased levels of DNA damage and reduced DNA repair capacity, most cancer cells accumulate numerous genomic alterations that differentiate them from normal cells.^11^ Although only a small subset of these genetic changes may account for tumor initiation, the overall landscape of DNA alterations provides important information regarding tumor DNA damage status and repair capacity and can confer the tumor with unique characteristics that have the potential to be exploited therapeutically. Interestingly, recent data have shown that DDR network has a major impact on the interaction between the tumor and the immune system.^12^

Standard treatment of HNSCC is a combination of surgery, radiotherapy and chemotherapy.^13^ The cytotoxic action of cisplatin is exerted through the development of DNA damage by the formation of intrastrand and interstrand cross-links and single-nucleotide damage of guanine. Nucleotide excision repair (NER) is the main process by which platinum intrastrand cross-links and single-nucleotide damage of guanine are repaired.^14^ The repair of interstrand cross-links requires a combination of NER, Fanconi anemia pathway, translesion synthesis and homologous recombination. Interestingly, interstrand cross-links repair proceeds via the formation of double-strand breaks (DSBs), the most lethal form of DNA damage.^15^, ^16^, ^17^

Poly(ADP-ribose) polymerase 1 (PARP1) is the founding member of an enzyme superfamily that serves to add poly(ADP) ribose moieties to target proteins and thus exert powerful effects on the repair of single-strand breaks (SSBs) and DSBs.^18^ There is a substantial level of evidence that tumor cells use PARP to repair platinum-induced DNA damage and thus escape apoptosis. To this end, the combination of platinum drugs with PARP inhibitors, such as the US-FDA- and EMA-approved olaparib, seems promising.^19^ During recent years, immune checkpoint blockade, using monoclonal antibodies targeting the PD-1/PD-L1 pathway, has led to impressive improvements in the effectiveness of immunotherapy against several cancers, including HNSCC.^20^ Since mounting evidence from trials in solid tumors suggests that alterations in DDR may predict response to immunotherapy,^12^ there is increasing rationale for combining PARP inhibition with immunotherapy. The defective DDR network can enhance the antitumor immune response in various ways. Deficiency in DNA damage repair causes accumulation of mutations resulting in increased tumor mutation burden and higher levels of major histocompatibility complex-presented neoantigens, which are recognized by T cells.^21^ Furthermore, failure of DDR increases cytosolic DNA, which binds to the cyclic guanosine monophosphate–adenosine monophosphate synthase and subsequently stimulates the innate immune response via the STING pathway.^22^, ^23^, ^24^, ^25^ Moreover, inhibition of ataxia-telangiectasia mutated induces an interferon-mediated innate immune response in a TANK Binding Kinase 1- and SRC-dependent manner.^26^ Together, these data support the hypothesis that tumors with underlying DNA repair defects may respond better to immune checkpoint inhibitors and also, targeting DDR can represent a relevant strategy to potentiate the efficacy of immune checkpoint inhibition.^27^, ^28^, ^29^, ^30^, ^31^, ^32^, ^33^, ^34^

We conducted a window of opportunity phase II trial (OPHELIA) in which patients were randomized (3 : 3 : 3 : 1) to receive cisplatin (60 mg/m^2^) on day 1 followed by olaparib (75 mg) on days 1-5, or olaparib (300 mg b.i.d.) for 21-28 days, or durvalumab (1500 mg) on day 1 followed by olaparib (600 mg) daily for 21-28 days or no treatment. Herein, we sought to determine whether DDR-related aberrations predict therapeutic benefit in cisplatin-treated HNSCC patients and how DDR pathways are modulated after treatment with olaparib.

## Patients and methods

### Patients

Peripheral blood mononuclear cells (PBMCs) from an annotated cohort of locally-advanced HNSCC patients treated with cisplatin-containing chemoradiation (35 responders, 8 non-responders), with nivolumab (*n* = 9) or enrolled in a window of opportunity phase II trial (OPHELIA, NCT02882308; olaparib alone, *n* = 3; cisplatin plus olaparib, *n* = 3; durvalumab plus olaparib, *n* = 12) were analyzed at diagnosis and at 24 h and 3 weeks following therapy (Supplementary Table S1, available at https://doi.org/10.1016/j.esmoop.2021.100075). PBMCs from healthy controls (HC; *n* = 46) were studied in parallel. All patients were staged according to the TNM (tumor–node–metastasis) staging of the American Joint Committee on Cancer (AJCC, 8th edition).^35^ Response assessment was based on RECIST 1.1 criteria.^36^ The study was approved by the Institutional Review Board of Attikon University Hospital, and all subjects provided informed consent. The study was conducted according to the Declaration of Helsinki. PBMCs were isolated from peripheral blood as described previously.^37^

### Cell lines

Human 1BR-3-hTert cells (immortalized normal skin fibroblasts)^38^ were maintained in Dulbecco's modified Eagle's medium (DMEM), supplemented with 10% fetal bovine serum (FBS) and 1% penicillin-streptomycin. Human GM12878 cells (B lymphoblastoid cells; Coriell Institute, Camden, New Jersey) were maintained in RPMI-1640 medium supplemented with 10% FBS and 1% penicillin-streptomycin. Human UM-SCC-11A cells (laryngeal squamous cell carcinoma cells; provided by Thomas Carey University of Michigan, Ann Arbor)^39^ were maintained in DMEM, supplemented with 1% non-essential amino acids, 10% FBS and 1% penicillin-streptomycin. Human CAL-33 cells (tongue squamous cell carcinoma cells),^40^ acquired from the Leibniz Institute DSMZ, Braunschweig, Germany (ACC 447), were maintained in DMEM supplemented with 2 mM glutamine, 10% FBS and 1% penicillin-streptomycin.

### Cell treatment

Cell lines were treated with 5 μg/ml cisplatin for 3 h at 37°C in culture medium. PBMCs were treated *ex vivo* with 5 μg/ml cisplatin for 3 h at 37°C in RPMI-1640 medium supplemented with 10% FBS, 100 units/ml penicillin, 100 mg/ml streptomycin and 2 mmol/l L-glutamine. Cells were subsequently incubated in drug-free medium for 0, 4, 8, 24 or 48 h, harvested and stored in freezing medium (90% FBS, 10% dimethyl sulfoxide) at −80°C.

### Single-cell gel electrophoresis (Comet assay)

Comet assay under alkaline or neutral conditions (Supplementary Materials and Methods, available at https://doi.org/10.1016/j.esmoop.2021.100075) was carried out as described.^41^

### Oxidative stress and abasic (apurinic/apyrimidinic) sites

Basal oxidative stress (Supplementary Materials and Methods, available at https://doi.org/10.1016/j.esmoop.2021.100075) was measured using a luminescence-based system that detects and quantifies total glutathione (GSH), oxidized glutathione [glutathione disulfide (GSSG)] and the GSH/GSSG ratio according to manufacturer's protocol (GSH/GSSG-Glo™ Assay, #V6612, Promega). Abasic sites (Supplementary Materials and Methods, available at https://doi.org/10.1016/j.esmoop.2021.100075) were evaluated using the OxiSelect Oxidative DNA Damage Quantitation Kit (Cell Biolabs; #STA-324) according to the manufacturer's protocol.

### Analysis of DSBs using confocal microscopy

Immunofluorescence antigen staining and confocal microscopy for the analysis of γH2AX foci (Cell Signaling Technology; #9718T) (Supplementary Materials and Methods, available at https://doi.org/10.1016/j.esmoop.2021.100075) was carried out as described.^42^

### Nucleotide excision repair

Cells were treated with 5 μg/ml cisplatin for 3 h at 37°C, resuspended in drug-free medium, incubated for 0, 8 and 24 h and harvested, and the cisplatin-induced DNA damage (Supplementary Materials and Methods, available at https://doi.org/10.1016/j.esmoop.2021.100075) was measured using Southern blot.^41^

### Cytotoxicity and cell proliferation

Cells were treated with 0-100 μg/ml cisplatin for 3 h, followed by 24 h, 48 h or 72 h post-incubation time in drug-free medium. The Cell Death Detection ELISA-PLUS kit (Roche Diagnostics, Switzerland) was used to determine apoptosis (Supplementary Materials and Methods, available at https://doi.org/10.1016/j.esmoop.2021.100075) according to the manufacturer's protocol.^42^

The analysis of drug-induced cytotoxicity and cell proliferation was carried out using the sulforhodamine B (SRB) assay.^43^ Cells were seeded for 24 h in 96-well microtiter plates. After treatment with 0, 10, 20, 30, 40 or 50 μg/ml cisplatin, fixation was carried out with 10% trichloroacetic acid and staining with 0.4% SRB in 1% acetic acid. Absorbance was measured using a microplate reader (TECAN, Switzerland) and cell viability was estimated.

### Expression of DDR-associated genes

PCR array analysis using the RT^2^ Profiler™ PCR array of 84 genes related to the DDR network (QIAGEN, #PAHS-029Z) (Supplementary Materials and Methods and Table S2, available at https://doi.org/10.1016/j.esmoop.2021.100075), were carried out as described.^44^ Two independent experiments were carried out for each sample.

### Statistical analysis

Continuous variables were compared among groups with Student's *t*-test, or Mann–Whitney *U* test when normal distribution did not apply, whereas paired comparisons were carried out by paired *t*-test or Wilcoxon's test. Correlations were examined with Spearman's rank test. Values are presented as mean ± standard deviation (SD). Independent associations were examined by multiple logistic regression analysis. Progression-free survival (PFS) was defined as the time from the date of start of treatment to the date of disease progression or death from other causes. Living patients who did not progress were censored at the last date they were progression-free. Survival curves were constructed according to the Kaplan–Meier method and PFS between groups was compared with log-rank test. Hazard ratios (HR) were calculated by Cox regression analysis. All tests were two-sided with a level of significance *P* < 0.05. All statistical analyses were carried out with SPSS v.24.0.

The study was conducted in accordance with the Declaration of Helsinki. The study protocol was approved by the Institutional Review Board of Attikon University Hospital.

## Results

### DDR signals in HNSCC cell lines

**Figure 1:**
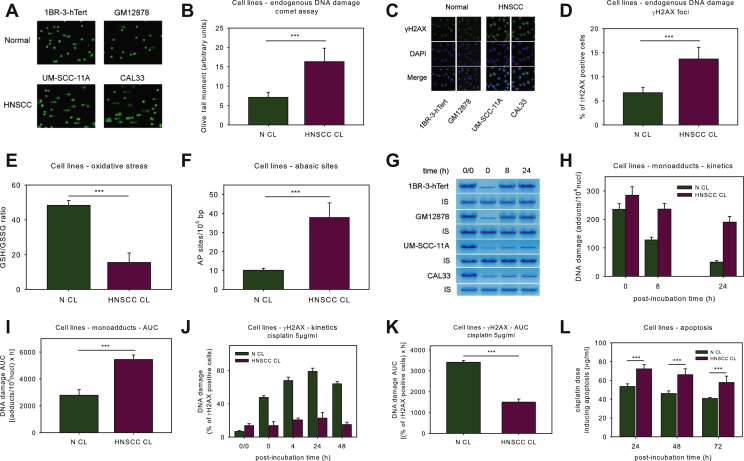
DNA damage response in HNSCC cell lines. (A) Alkaline comet assay images of normal and HNSCC cell lines. (B) Bar charts showing distribution of the endogenous DNA damage measured by comet assay. (C) Typical images showing γH2AX staining using confocal microscopy. Upper images, γH2AX staining; middle, cell nuclei labeled with DAPI; bottom, merged. (D) Bar charts showing distribution of the endogenous DSBs measured by γH2AX staining. Bar charts showing distribution of (E) the oxidative stress and (F) AP-sites. (G) Southern blot of the cisplatin-induced N-ras-specific monoadducts in normal and HNSCC cell lines. Bar charts showing (H) the kinetics of monoadducts repair and (I) the accumulation of monoadducts following cisplatin treatment. N CL, normal cell line; HNSCC CL, HNSCC cell line. Bar charts showing (J) the kinetics of γH2AX foci formation/removal and (K) the accumulation of γH2AX foci after cisplatin treatment. (L) Bar charts showing distribution of the lowest concentrations of cisplatin required for the induction of apoptosis 24 h, 48 h and 72 h after cisplatin treatment. Error bars represent SD; ∗∗∗*P* < 0.001 by Mann–Whitney *U* test. The experiments shown were based on a minimum of three independent repeats. AP, abasic sites; AUC, area under the curve; CL, cell line; DAPI, 4′,6-diamidino-2-phenylindole; DSBs, double-strand breaks; HNSCC, head and neck squamous cell cancer; IS, internal standard; N CL, normal cell line; SD, standard deviation.

DDR signals were analyzed in HNSCC (UM-SCC-11A, CAL33) and normal (1BR-3-hTert, GM12878) cell lines. For all endpoints analyzed, similar results were obtained for the cell lines of each pair. Firstly, the presence of endogenous DNA damage was assessed using an alkaline comet assay measuring SSBs and/or DSBs. As seen in Figure 1A and B, the levels of endogenous DNA damage were found to be significantly higher in HNSCC cells compared with normal ones (*P* < 0.001), indicating accumulation of DNA damage in malignant cells in the absence of known exogenous genotoxic insults. Next, significantly elevated levels of γH2AX foci were observed in HNSCC cells (*P* < 0.001; Figure 1C and D), confirming the accumulation of DSBs in these cells.

To investigate the formation of DNA damage, we evaluated critical factors/processes that lead to intracellular formation of SSBs and DSBs, namely oxidative stress and apurinic/apyrimidinic (AP) sites. Compared with normal cells, HNSCC cells showed significantly higher levels of both oxidative stress (as indicated by the reduction of the GSH/GSSG ratio; Figure 1E) and AP-sites (Figure 1F) (all *P* < 0.001), suggesting that the increased endogenous DNA damage measured in malignant cells may result, at least in part, from oxidative stress and/or the induction of AP-sites.

Then, the efficiency of NER was measured using Southern blot. For this purpose, we treated cells with 5 μg/ml cisplatin, and the repair kinetics of NER-repaired monoadducts was followed at the N-ras gene.^45^ Much lower rates of NER were found in HNSCC than normal cells (Figure 1G and H), resulting in significantly higher DNA damage burden, expressed as the area under the curve (AUC) for DNA adducts during the whole experiment, in malignant cells (*P* < 0.001) (Figure 1I).

To study the repair of DSBs, cells were treated with 0, 2.5, 5, 10, 25 or 50 μg/ml cisplatin, and γH2AX foci levels were examined. HNSCC cells showed significantly higher DSB repair capacity compared with normal ones, resulting in lower DSBs burden in malignant cells (*P* < 0.001; Figure 1J and K and Supplementary Figure S1, available at https://doi.org/10.1016/j.esmoop.2021.100075). Similar results were obtained using neutral comet assay measuring DSBs only (cisplatin doses: 25, 50 μg/ml; Supplementary Figure S2, available at https://doi.org/10.1016/j.esmoop.2021.100075).

The apoptosis rates of HNSCC cells were evaluated 24 h, 48 h and 72 h after treatment with 0-100 μg/ml cisplatin. At all time-points, the lowest cisplatin concentrations required for the induction of apoptosis were significantly higher in HNSCC cells than normal ones (all *P* < 0.001; Figure 1L), indicating that malignant cells exhibit significantly reduced apoptosis rates. Similar results were obtained using the SRB assay, with malignant cells showing higher cisplatin resistance (cisplatin doses: 0, 10, 20, 30, 40, 50 μg/ml; Supplementary Figure S3, available at https://doi.org/10.1016/j.esmoop.2021.100075).

### DDR signals in PBMCs from HNSCC patients at baseline

**Figure 2:**
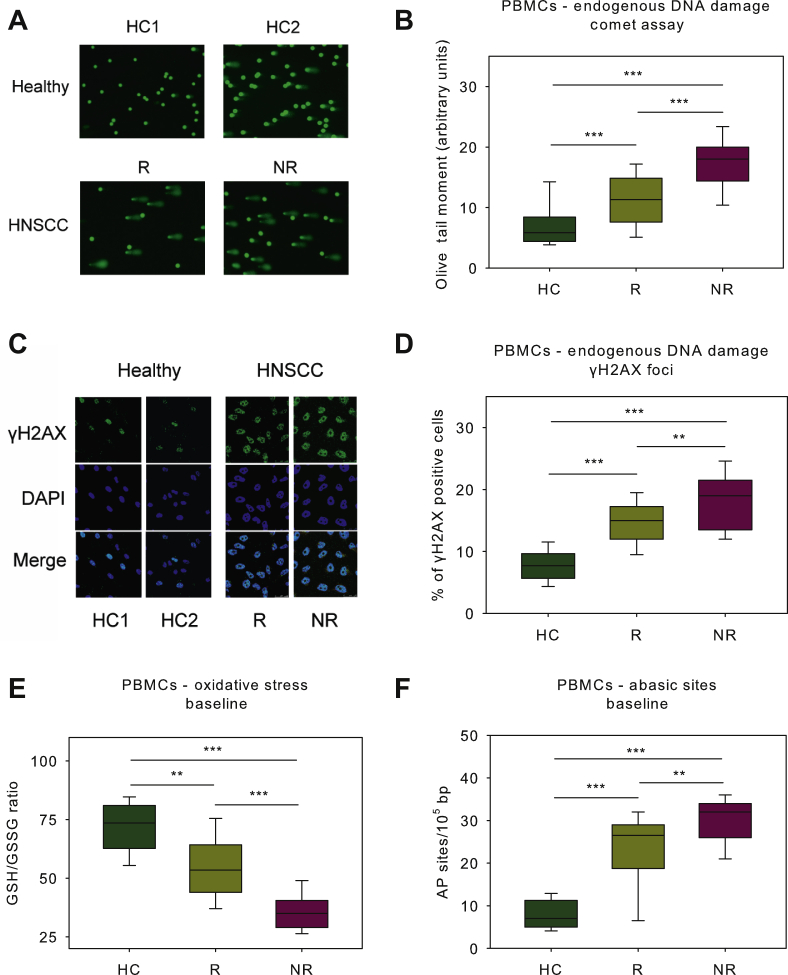
Endogenous DNA damage in PBMCs from HNSCC patients. (A) Alkaline comet assay images of two healthy controls (HC1, HC2) and two HNSCC patients at baseline. (B) Box plots showing statistical distribution of the endogenous DNA damage measured by comet assay in HC and HNSCC patients at baseline. (C) The immunofluorescence γH2AX staining of two HC and two HNSCC patients. (D) Box plots showing statistical distribution of the endogenous DNA damage in HC and patients at baseline. Box plots showing statistical distribution of the oxidative stress (E) and the AP-sites (F) in HC and patients at baseline. Error bars represent SD; ∗∗∗*P* < 0.001 by Mann–Whitney *U* test. The experiments shown were based on a minimum of three independent repeats. AP, abasic sites; DAPI, 4′,6-diamidino-2-phenylindole; HC, healthy control; HNSCC, head and neck squamous cell cancer; PBMCs, peripheral blood mononuclear cells; NR, non-responder; R, responder; SD, standard deviation.

To determine whether the cell lines findings extend to patient-derived samples, changes in critical DDR signals were evaluated in PBMCs from 43 HNSCC patients at baseline (35 responders and 8 non-responders to subsequent cisplatin-containing chemoradiation). Forty-six healthy individuals served as controls. In accordance with the results from the cell lines experiments, patients at baseline showed significantly higher levels of endogenous DNA damage compared with HC, with cisplatin non-responders showing the highest levels (Figure 2A-D; all *P* < 0.05).

Furthermore, the formation of DNA damage was evaluated. We found that patients exhibited significantly higher levels of oxidative stress (Figure 2E) and AP-sites (Figure 2F; all *P* < 0.001) than HC, with cisplatin non-responders showing again the highest levels. In accordance with previous studies showing that oxidative stress produces AP-sites,^46^ significant correlation was observed between individual oxidative stress and AP-sites (r = −0.746, *P* < 0.001; Supplementary Figure S4A, available at https://doi.org/10.1016/j.esmoop.2021.100075). Also, significant correlations were found (i) between oxidative stress and DNA damage levels (comet: r = −0.916, *P* < 0.001; γH2AX: r = −0.796, *P* < 0.001; Supplementary Figure S4B and C, available at https://doi.org/10.1016/j.esmoop.2021.100075), and (ii) between AP-sites and DNA damage levels (comet: r = 0.727, *P* < 0.001; γH2AX: r = 0.694, *P* < 0.001; Supplementary Figure S4D and E, available at https://doi.org/10.1016/j.esmoop.2021.100075), suggesting that the increased endogenous DNA damage observed in patients may result, at least in part, from oxidative stress and/or the induction of AP-sites.

### DDR signals in PBMCs from HNSCC patients following ex vivo treatment

**Figure 3:**
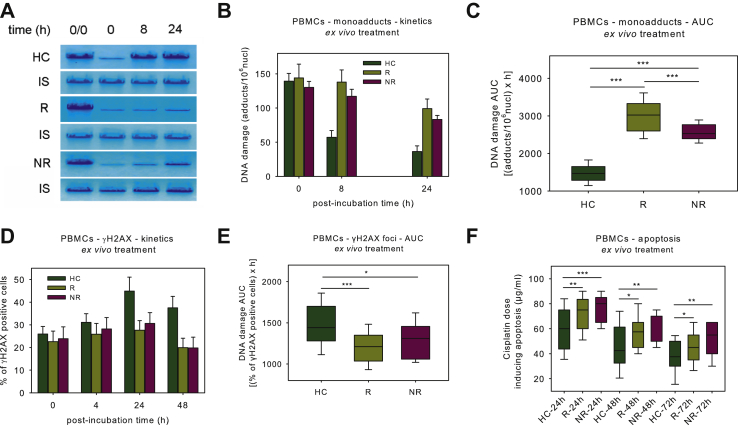
DDR signals in PBMCs following *ex vivo* cisplatin treatment. (A) Southern blot of the *ex vivo* cisplatin-induced monoadducts in a HC and two HNSCC patients (R, responder; NR, non-responder). (B) The kinetics of monoadducts repair and (C) the statistical distribution of the accumulation of the *ex vivo* cisplatin-induced monoadducts, in HC and HNSCC patients at baseline. (D) The kinetics of γH2AX foci and (E) the statistical distribution of the *ex vivo* cisplatin-induced γH2AX foci accumulation in HC and HNSCC patients. (F) Box plots showing statistical distribution of the lowest concentrations of cisplatin required for the induction of apoptosis 24 h, 48 h and 72 h after *ex vivo* treatment with cisplatin. Error bars represent SD; ∗*P* < 0.05, ∗∗*P* < 0.01 and ∗∗∗*P* < 0.001 by Mann–Whitney *U* test. The experiments shown were based on a minimum of three independent repeats. AUC, area under the curve; DDR, DNA damage response; HC, healthy control; HNSCC, head and neck squamous cell cancer; IS, internal standard. NR, non-responder; PBMCs, peripheral blood mononuclear cells; R, responder.

The efficiencies of NER and DSB repair were also analyzed in PBMCs at baseline after *ex vivo* treatment with 5 μg/ml cisplatin. In line with the cell lines' results, patients showed lower NER capacity than HC, with cisplatin responders showing the lowest levels (Figure 3A and B). Significantly higher accumulation of NER-repaired DNA damage was observed in patients' cells, with responders showing the highest levels (all *P* < 0.001; Figure 3C). Higher rates of DSB repair were observed in patients than HC (Figure 3D), resulting in significantly reduced DSBs burden in patients' cells (all *P* < 0.05; Figure 3E). Similar DSB repair activities were observed in both responders and non-responders patients.

The induction of apoptosis was evaluated 24 h, 48 h and 72 h after *ex vivo* treatment with 0-100 μg/ml cisplatin. At all time-points, significantly reduced apoptosis rates were observed in patients' PBMCs compared with HC (all *P* < 0.05; Figure 3F). Although cisplatin non-responders showed lower apoptosis rates than responders, the difference did not reach statistical significance. Interestingly, Mann–Whitney *U* test showed that differences between individual DDR parameters at baseline (endogenous DNA damage, DSB repair capacity, oxidative stress, abasic sites) observed in PBMCs are not affected by patients' characteristics, including age, sex, excess alcohol intake and smoking history (Supplementary Table S3 and Supplementary Figures S5-S8, available at https://doi.org/10.1016/j.esmoop.2021.100075).

### Correlation between individual DDR signals at baseline and PFS with cisplatin-based therapy

**Figure 4:**
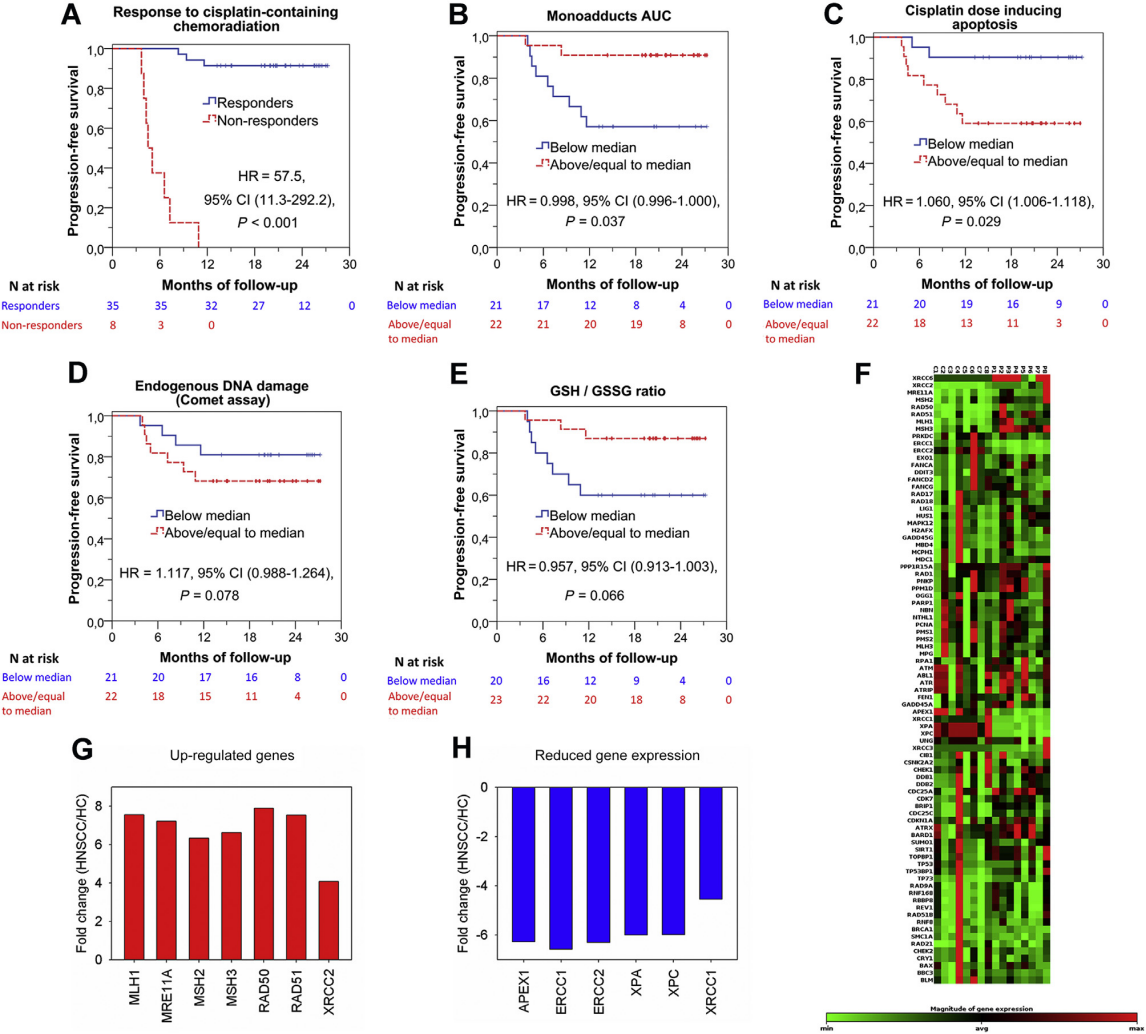
Kaplan–Meier PFS curves and expression of DDR-associated genes. (A) Kaplan–Meier PFS curves according to response to cisplatin-containing chemoradiation. Kaplan–Meier curves demonstrating that longer PFS is associated with (B) lower NER resulting in higher DNA damage burden, (C) increased apoptosis, (D) lower endogenous DNA damage, and (E) decreased oxidative stress at baseline. (F) Hierarchical clustergram of 84 DDR-associated genes in eight HNSCC patients at baseline versus eight HC. (G, H) Genes demonstrating at least two-fold difference in the transcription activity between patients and HC. AUC, area under the curve; CI, confidence interval; DDR, DNA damage response; GSH, glutathione; GSSG, glutathione disulfide; HC, healthy control; HNSCC, head and neck squamous cell cancer; HR, hazard ratio; NER, nucleotide excision repair; PFS, progression-free survival.

The 43 HNSCC patients treated with cisplatin-containing chemoradiation were followed for a median of 20.8 months (range, 5.3-27.2). During this time, 11 patients progressed and 4 died. A Kaplan–Meier survival curve of responders and non-responders to cisplatin/radiotherapy is shown in Figure 4A. We found that lower NER capacity at baseline resulting in higher drug-induced DNA damage burden, expressed as the AUC for DNA adducts, was associated with better PFS [HR = 0.998, 95% confidence interval (CI) (0.996-1.000), *P* = 0.037; Figure 4B]. Also, reduced apoptosis rates at baseline, expressed as the lowest concentration of cisplatin required for the induction of apoptosis, was associated with shorter PFS [HR = 1.060, 95% CI (1.006-1.118), *P* = 0.029; Figure 4C]. Of note, the Cox proportional hazard model demonstrated the independent prognostic significance for PFS of NER capacity and apoptosis rates at baseline (Supplementary Tables S4 and S5, available at https://doi.org/10.1016/j.esmoop.2021.100075). Although higher endogenous DNA damage was also associated with shorter PFS [HR = 1.117, 95% CI (0.988-1.264), *P* = 0.078; Figure 4D] and decreased oxidative stress at baseline with longer PFS [HR = 0.957, 95% CI (0.913-1.003), *P* = 0.066; Figure 4E], the results did not reach statistical significance.

### Expression of DDR-associated genes in PBMCs

The expression of DDR-associated genes was analyzed in PBMCs from eight patients at baseline and eight HC (Figure 4F). Of 84 genes examined (Supplementary Table S2, available at https://doi.org/10.1016/j.esmoop.2021.100075), a total of 13 genes representing several non-mutually exclusive categories demonstrated at least two-fold difference in gene expression between patients and HC. Particularly, DSB repair (*MRE11A, RAD50, RAD51, XRCC2*) and mismatch repair (MMR; *MLH1, MSH2, MSH3*) genes were up-regulated in patients versus HC (Figure 4G), whereas expression of NER (*ERCC1, ERCC2/XPD, XPA, XPC*) and base excision repair (BER; *APEX1, XRCC1*) genes was reduced (Figure 4H).

### DDR signals in PBMCs from HNSCC patients following in vivo/therapeutic treatment

**Figure 5:**
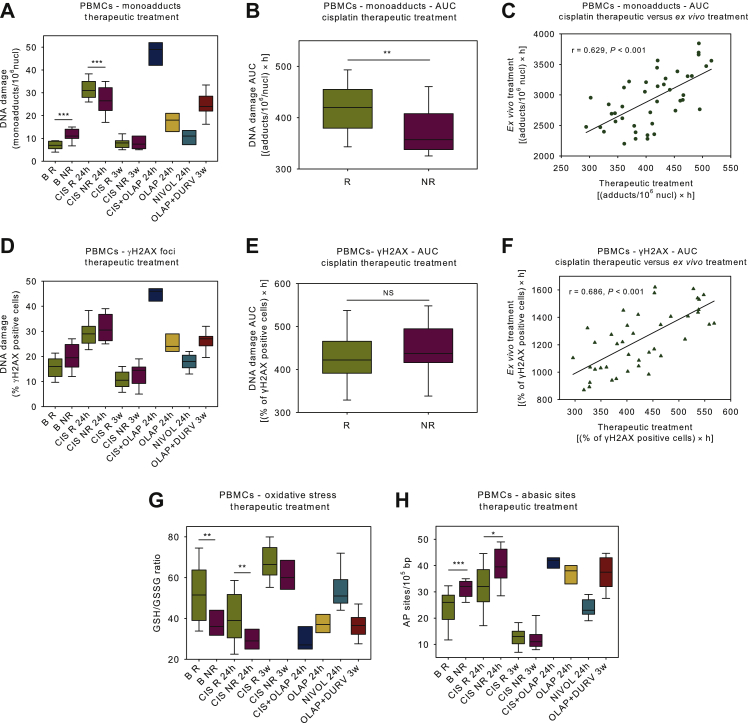
DDR signals following various *in vivo*/therapeutic treatments. (A) Box plots showing the kinetics of monoadducts repair in patients following various therapeutic treatments. (B) Box plots showing statistical distribution of the accumulation of monoadducts after cisplatin-only therapy. (C) Correlation between monoadducts burden following therapeutic and *ex vivo* cisplatin treatment in the same patients. (D) Box plots showing the kinetics of γH2AX foci formation/removal following various therapeutic treatments. (E) Box plots showing the statistical distribution of the accumulation of γH2AX foci after cisplatin-only therapy. (F) Correlation between DSB burden following therapeutic and *ex vivo* cisplatin treatment in the same patients. Box plots showing statistical distribution of the oxidative stress (G) and the abasic sites (H) after various therapeutic treatments. Error bars represent SD; ∗*P* < 0.05, ∗∗*P* < 0.01 and ∗∗∗*P* < 0.001 by Mann–Whitney *U* test. The experiments shown were based on a minimum of three independent repeats. AUC, area under the curve; DDR, DNA damage response; DSB, double-strand break; GSH, glutathione; GSSG, glutathione disulfide; NR, non-responder; NS, not significant; PBMCs, peripheral blood mononuclear cells; R, responder; SD, standard deviation.

The effect of cisplatin therapy on DDR signals was also analyzed. Peak monoadduct levels were found within 24 h following drug treatment (Figure 5A). In accordance with the *ex vivo* data from the same patients, cisplatin responders showed lower NER capacity than non-responders, resulting in the accumulation of higher monoadducts burden in responders' cells (Figure 5A and B; *P* < 0.01). Significant correlation was observed in the individual monoadducts burden between therapeutic and *ex vivo* treated cells from the same patients (Figure 5C; r = 0.629, *P* < 0.001). Maximal DSBs levels were observed 24 h after therapeutic treatment in all patients analyzed, with DSB repair efficiency being similar in both responders and non-responders patients (Figure 5D and E). Significant correlation was observed in individual DSBs' burden between therapeutic and *ex vivo* treatment (Figure 5F; r = 0.686, *P* < 0.001). Both oxidative stress and AP-sites showed maximal levels 24 h after cisplatin treatment, with cisplatin non-responders showing significantly higher levels than responders (Figure 5G and H; *P* < 0.01). Three weeks after treatment, oxidative stress and AP-sites decreased to baseline levels.

Moreover, we found that following therapeutic treatment with olaparib-containing regimens NER-repaired monoadducts (Figure 5A), DSBs (Figure 5D), oxidative stress (Figure 5G) and AP-sites (Figure 5H) were significantly increased compared with baseline levels (all *P* < 0.05). Notably, olaparib plus cisplatin therapy showed significantly higher DNA damage levels (both monoadducts and DSBs) compared with cisplatin-only treatment, possibly due to olaparib-induced inhibition of DNA repair.^18^ Nivolumab induced no significant changes in the DDR parameters examined.

## Discussion

Endogenous DNA damage poses a serious threat to cell fate since it may lead to mutagenesis, genomic instability and cellular apoptosis.^47^ Herein, increased endogenous DNA damage was observed in PBMCs from HNSCC patients compared with HC, with cisplatin non-responders showing the highest levels. To understand the origin of the increased endogenous DNA damage, we evaluated the induction of oxidative stress and AP-sites.^46^ Both of these factors/processes were found to be significantly higher in HNSCC patients at baseline compared with HC. Accordingly, previous studies have shown that HNSCC bears a strong link to oxidative stress since tobacco and alcohol, which are known to increase ROS production, are clearly defined as etiological factors for this malignancy.^48^ The excessive oxidative stress of HNSCC patients could also explain, at least in part, the increased levels of AP-sites and DSBs that were found in our patients since ROS produce such types of DNA damage.^49^ Also, we found that decreased oxidative stress is associated with longer PFS. In line with our results, recent data have shown that HNSCC patients with lower oxidative stress had a lower risk of local and regional recurrence of tumor after treatment, suggesting a more aggressive behavior of tumors with high oxidative stress.^50^

The efficiency of fundamental DNA repair mechanisms, namely NER and DSB repair, were also evaluated. NER eliminates a broad spectrum of DNA lesions, including those produced by ROS and DNA adducts formed by tobacco smoking and cancer therapeutic drugs such as cisplatin.^51^ Our previous studies have shown that the efficiency of NER is critical for cell survival,^37^^,^^44^ and is associated with drug-response to therapeutic treatment.^42^^,^^52^ Herein, HNSCC patients showed decreased NER capacity compared with HC, with cisplatin responders showing lower NER activity than non-responders. These results suggest that NER status is implicated in both HNSCC pathogenesis and resistance to genotoxic drugs. In accordance with these results, Sliwinski et al.^53^ have shown significantly decreased NER capacity of PBMCs from HNSCC patients than HC. Accordingly, we found that critical NER-associated genes (*ERCC1, ERCC2/XPD, XPA, XPC*) were downregulated in HNSCC patients compared with HC, thus explaining in part the reduced NER capacity of these patients. Other reports have also shown that polymorphisms in NER genes (*XPA, XPC, ERCC2/XPD, ERCC1*) are implicated in the onset and progression of HNSCC, as well as the response to therapy.^54^^,^^55^ Interestingly, we found that lower NER capacity correlated with longer PFS, suggesting that although interstrand cross-links and DSBs are thought to be the main determinants of cisplatin toxicity,^14^ the repair of DNA monoadducts before crosslinking and the subsequent formation of DSBs may play an important role in protecting cells from cisplatin cytotoxicity and may be a significant factor leading to chemotherapy failure. Similar results were obtained in our previous study on multiple myeloma patients who undergo melphalan therapy,^56^ suggesting that quantitation of NER capacity at baseline may identify patients who are more likely to benefit from such treatment.

Previous studies have reported that constitutively activated DSB repair capacity may facilitate the acquisition and progression of a tumor and/or the induction of a drug-resistant phenotype.^57^ In agreement with these data, we found increased DSB repair capacity of HNSCC patients compared with HC. Moreover, we found that critical DSB repair-associated genes (*MRE11A, RAD50, RAD51, XRCC2*) were overexpressed in HNSCC patients compared with HC, thus explaining in part the increased DSB repair capacity of these patients. Previous studies have reported that polymorphisms in DSB repair genes (*RAD51, MRE11A, XRCC2, XRCC3*) are implicated in the risk of predisposition to HNSCC.^58^^,^^59^ Moreover, we found that several MMR-related genes (*MLH1, MSH2, MSH3*) were overexpressed, while expression of BER-associated genes (*APEX1, XRCC1*) was reduced in HNSCC patients compared with HC. Previous studies also showed that polymorphisms in MMR (*MLH1, MSH2, MSH3*)^60^ and BER genes (*APEX1, XRCC1*)^61^ may contribute to the progression of HNSCC.

Also, we observed lower apoptosis rates in HNSCC patients than HC, a finding that is in agreement with previous studies showing that evasion of apoptosis is a hallmark of cancer.^62^ Indeed, the loss of apoptotic control allows cancer cells to survive longer and gives more time for the accumulation of mutations, which can increase invasiveness during tumor progression, stimulate angiogenesis, deregulate cell proliferation and interfere with differentiation.^63^ Interestingly, we found that increased apoptosis of PBMCs at baseline was associated with better PFS. Corresponding results on all DDR signals examined were observed in HNSCC cell lines, thus confirming the generality of our findings.

Importantly, we found that in addition to compromising the DNA repair, olaparib may exert extra antitumor effect by elevating oxidative stress, which further results in the induction of DNA damage.^64^^,^^65^ Since PARP inhibition-induced DNA damage enhances immune response and PD-L1 expression,^66^ there is increased rationale for PARP/anti-PD-1 combination. Along with new therapeutic options, accurate and predictive biomarkers should be found and established. Homologous recombination deficiency and immune inflammation indicators, i.e. STING and interferon signatures may be the ideal candidates to predict benefit from combination of PARP inhibitors with chemotherapy or immunotherapy. Elucidating the interplay between various biomarkers with respect to activity will be critical to the future development of such combinations and necessitates the integration of precision oncology during early clinical trial setting. For example, the definition of DDR deficiency will likely be very important to optimize patient selection. Obtaining serial tumor biopsies and matched blood samples is critical to fully define not only the presence of DDR defects, but also their nature. For instance, are they germline or somatic, and if somatic, are they early, relatively clonal or late subclonal events? It will also be important to understand the effects of different forms of DDR defects on tumor immunogenicity. To this end, it will be critical to combine genomic profiling with sequencing of the T-cell receptor, gene expression profiling and immunohistochemistry/fluorescent assessments of PD-L1 expression and broad immune infiltrate in order to understand the relationship between DDR and immune-related biomarker groups and to generate a deeper understanding of how DNA damage interacts with antitumor immune response.

One of the limitations of our study is the heterogeneity of enrolled patients in terms of stage and treatment. Our findings did not retain significance in multivariate analysis due to small sample size. The DDR biomarkers were assessed in relation to therapeutic response in cisplatin-treated patients only. Validation of our results in future studies with larger cohorts is needed. Moreover, since DDR parameters were examined in cell lines and PBMCs, another limitation of the present study is that it is not clear if DDR signals that occur in tumor tissues from HNSCC patients are reflected in PBMCs. Of note, previous studies in solid tumors have shown that monitoring of DDR parameters in the blood may be potentially useful in patient prognosis^53^^,^^67^, ^68^, ^69^, ^70^, ^71^, ^72^, ^73^, ^74^ and prediction of treatment response.^52^^,^^75^, ^76^, ^77^, ^78^, ^79^, ^80^, ^81^, ^82^, ^83^, ^84^, ^85^ Therefore, a key challenge for our future research is to examine DDR parameters in both tumor biopsies and PBMCs from the same HNSCC patients.

In conclusion, changes in DDR signals are implicated in the response to HNSCC chemotherapy and can be exploited as novel therapeutic targets and sensitive/effective non-invasive biomarkers. Interestingly, since deficiencies in DNA repair mechanisms have been found to shape the immune response, these results may lead to identification of DDR-based tools for the selection of immunotherapy candidates.
