# Supplementary material for: The DNA damage response network in the treatment of head and neck squamous cell carcinoma
Source: ESMO Open. 2021 Mar 10;6(2):100075. doi: 10.1016/j.esmoop.2021.100075 (PMC7957155; doi:10.1016/j.esmoop.2021.100075)
Supplement: Supplementary Material [file mmc1.doc]

**SUPPLEMENTARY DATA**

**The DNA damage response network in the treatment of Head and Neck Squamous Cell Carcinoma**

**Table of Contents**

- Supplementary Materials and Methods
- Supplementary Table S1. Patients and disease characteristics
- Supplementary Table S2. RT² Profiler™ PCR Array Human DNA Damage Signaling Pathway: Gene list
- Supplementary Table S3. Correlations between individual DDR parameters at baseline and patients’ characteristics using Mann-Whitney U test
- Supplementary Table S4. Cox proportional hazard model demonstrating the independent prognostic significance for progression-free survival of NER capacity (expressed as DNA damage AUC)
- Supplementary Table S5. Cox proportional hazard model demonstrating the independent prognostic significance for progression-free survival of apoptosis (expressed as cisplatin dose inducing apoptosis)
- Supplementary Figure S1. DSB formation and repair using immunofluorescence γH2AX staining and confocal microscopy in cell lines
- Supplementary Figure S2. DSB formation and repair using neutral comet assay in cell lines
- Supplementary Figure S3. Cytotoxicity and cell proliferation in cell lines
- Supplementary Figure S4. Correlations between the endogenous DNA damage, oxidative stress and abasic sites in PBMCs at baseline
- Supplementary Figure S5. Correlations between endogenous DNA damage and patients’ characteristics using Mann-Whitney U test
- Supplementary Figure S6. Correlations between DSB repair capacity at baseline and patients’ characteristics using Mann-Whitney U test
- Supplementary Figure S7. Correlations between oxidative stress at baseline and patients’ characteristics using Mann-Whitney U test
- Supplementary Figure S8. Correlations between abasic sites at baseline and patients’ characteristics using Mann-Whitney U test

**SUPPLEMENTARY MATERIALS AND METHODS**

**Single-cell gel electrophoresis (comet assay)**

An appropriate cell suspension (40µl) was diluted in 400µl of 0.5% low-melting point agarose and 75µl were pipetted on to glass slides precoated with 1% high-melting point agarose. After lysis (performed in prechilled lysis solution; Trevigen), slides were placed in a comet assay tank (Trevigen Inc., Gaithersburg, MD) and incubated in prechilled electrophoresis buffer (alkaline or neutral, as indicated). Electrophoresis was performed at 21V, 300mA for 30min in the comet assay tank at 4^o^C. Afterward, slides were washed in prechilled deionized H_2_O, fixed in 70% ethanol and stained with SYBR™ Gold nucleic acid gels stain (Thermo Fisher Scientific, #S11494). Comet parameters were analyzed by CometScore (TriTek Corp, Sumerduck, VA).

**Measurement of glutathione (GSH) and oxidized glutathione (GSSG)**

Basal oxidative stress was measured using a luminescence-based system that detects and quantifies total glutathione, oxidized glutathione and the GSH/GSSG ratio according to manufacturer’s experimental protocol (GSH/GSSG-Glo™ Assay, Promega). Briefly, 10^4^ cells were plated in 96-well luminometer-compatible tissue culture plate (Corning Costar). Afterwards, 50μl/well of Luciferin Generation Reagent was added, followed by a brief shake and incubation at room temperature for 30min. Subsequently, 100μl/well of Luciferin Detection Reagent was added and after 15min incubation the luminescence signal was read in Spectramax M3 microplate reader (Molecular Devices LLC, California).

**Detection of abasic sites**

Abasic sites were evaluated using the OxiSelect Oxidative DNA Damage Quantitation Kit (AP-sites) according to manufacturer’s experimental protocol (Cell Biolabs, Inc.). The assay kit uses an Aldehyde Reactive Probe to react specifically with an aldehyde group on the open ring form of AP-sites. This allows for the AP-sites to be tagged with biotin, which is later detected with Streptavidin-Enzyme conjugate. The quantity of AP-sites in unknown DNA sample is determined by comparing its absorbance with a standard curve generated from the provided DNA standard containing predetermined AP-sites.

**Immunofluorescence antigen staining and confocal laser scanning microscope analysis**

Aliquots of 2x10^4^ cells were adhered to coverslip coated with 1M HCI and 50mg/ml poly-D-lysine prior to use, fixed by adding a 4% paraformaldehyde solution for 6min at room temperature and stored at -70°C until the analysis of γH2AX. Cells were washed with cold PBS and blocked with 0.5ml per well blocking buffer (0.1% Triton X-100, 0.2% skimmed dry milk in PBS) for 1h at room temperature in a humidified box. Blocked cells were incubated with antibody against γH2AX (Cell Signaling Technology, #9718T) in blocking buffer at 4°C overnight. After washing with blocking buffer, cells were incubated with fluorescent secondary antibody (Abcam; AlexaFluor 488 goat anti-mouse IgG; #ab150113) and images were visualized with a Leica TCS SP-1 confocal laser scanning microscope (Leica, Wetzlar, Germany). Foci were manually counted in 200 cells/treatment condition and results are expressed as the % of γH2AX positive cells (mean±SD); positive cells are defined as cells with more than 5 foci per cell.

**Nucleotide Excision Repair**

For the measurement of gene-specific monoadducts, following DNA isolation, genomic DNA was digested to completion with EcoRI and DNA samples dissolved in sterile deionized H_2_O were heated at 70^o^C for 30min to depurinate N-alkylated bases. Apurinic sites were converted to single-strand breaks by the addition of NaOH for 30min at 37^o^C, size fractionated using agarose gel electrophoresis, and Southern blotted. Hybridizations were performed using a 112-bp PCR fragment (forward primer, 5’-GTT-ATA-GAT-GGT-GAA-ACC-TG-3’; reverse primer, 5’-ATA-CAC-AGA-GGA-AGC-CTT-CG-3’). Following exposure, band volumes were determined using a Molecular Dynamics Personal Densitometer. The average frequency of monoadducts in the restriction fragment of interest (N-ras gene) was calculated from the fraction of DNA in the band of the treated sample as compared to that from the non-treated sample. Assuming the random introduction of monoadducts in a homogeneous population of fragments, we applied the Poisson expression to calculate the average number of monoadducts per restriction fragment: (adducts/fragment)=-log_e_(fraction of fragment free of adducts).

**Apoptosis assay**

Cells (2x10^4^ cells) were treated with 0-100μg/ml of cisplatin for 3h, followed by 24h, 48h or 72h post-incubation time in drug-free medium. Apoptosis was evaluated by using the Cell Death Detection ELISA-PLUS kit (Roche Applied Sciences), according to manufacturer’s instructions. Briefly, cells were collected to prepare the cytosolic fractions that contained fragments of DNA. Equal volumes of these cytosolic fractions were incubated in anti-histone antibody-coated wells (96-well plates), and the histones of the DNA fragments were allowed to bind to the anti-histone antibodies. The peroxidase-labeled mouse monoclonal DNA antibodies were used to localize and detect the bound fragmented DNA using photometric detection with 2,2′-azino-di-(3-ethylbenzthiazoline sulfonate) as the substrate. The test quantifies apoptosis as the fold increase (expressed as enrichment factor, EF) in the level of apoptosis in treated samples to untreated samples. That is, we calculated the specific enrichment of mono- and oligo-nucleosomes released into the cytoplasm using the following formula: (EF)=(absorbance of the drug-treated cells)/(absorbance of the cells without drug treatment). Finally, the individual apoptosis rate following drug treatment was expressed as the drug dose sufficient to trigger the induction of a certain enrichment factor (EF=3).

**RT^2^ Profiler™ PCR Array**

Total RNA was extracted using the Qiagen RNeasy Mini Kit according to manufacturer’s instructions and stored at -80^o^C until use. For PCR array analysis, the RT² Profiler™ PCR Array Human DNA Damage Signaling Pathway (QIAGEN; #PAHS-029Z) was utilized. Total RNA (200ng) was reverse transcribed using the RT^2^ First Strand kit following the manufacturer’s instructions. Resulting cDNA was diluted in nuclease-free water and added to RT^2^ SYBR Green Fluor qPCR Mastermix (QIAGEN) which was subsequently aliquoted to each well of the PCR array for quantitative PCR. Thermal cycling and fluorescence detection were performed using a Biorad iQ™5 (Bio-Rad Laboratories, UK). Cycling conditions were: 10min denaturation at 95˚C followed by 40 cycles of 15sec at 95˚C and 1min at 60˚C. Data were analyzed using the RT^2^ Profiler PCR Array Data Analysis Webportal (<https://geneglobe.qiagen.com/gr/analyze/>).

| **Supplementary Table S1. Patients and disease characteristics** | | | | | | |
| --- | --- | --- | --- | --- | --- | --- |
| **Characteristics** | | | **Response to treatment** | | **Total** | ***P*-value** |
|  |  |  | **Response** | **No response** |  |  |
| Age | <60 years | N | 19 | 7 | 26 | 0.704 |
|  |  | % | 27.2% | 10% | 37.2% |  |
|  | ≥60 years | N | 22 | 22 | 44 |  |
|  |  | % | 31.4% | 31.4% | 62.8% |  |
| Sex | Female | N | 7 | 7 | 14 | 0.153 |
|  |  | % | 10% | 10% | 20% |  |
|  | Male | N | 34 | 22 | 56 |  |
|  |  | % | 48.6% | 31.4% | 80% |  |
| Primary | Oral cavity | N | 15 | 13 | 28 | 0.054 |
|  |  | % | 21.4% | 18.6% | 40% |  |
|  | Oropharynx | N | 18 | 6 | 24 |  |
|  |  | % | 25.7% | 8.6% | 34.3% |  |
|  | Larynx | N | 8 | 10 | 18 |  |
|  |  | % | 11.4% | 14.3% | 25.7% |  |
| Alcohol use | Heavy | N | 17 | 12 | 29 | 0.876 |
|  |  | % | 24.3% | 17.1% | 41.4% |  |
|  | Social | N | 6 | 7 | 13 |  |
|  |  | % | 8.6% | 10% | 18.6% |  |
|  | No | N | 12 | 8 | 20 |  |
|  |  | % | 17.1% | 11.5% | 28.6% |  |
|  | N/A | N | 6 | 2 | 8 |  |
|  |  | % | 8.6% | 2.8% | 11.4% |  |
| Tobacco use | Current | N | 18 | 11 | 29 | 0.762 |
|  |  | % | 25.7% | 15.7% | 41.4% |  |
|  | Former | N | 13 | 12 | 25 |  |
|  |  | % | 18.6% | 17.1% | 35.7% |  |
|  | No | N | 7 | 5 | 12 |  |
|  |  | % | 10% | 7.1% | 17.1% |  |
|  | N/A | N | 3 | 1 | 4 |  |
|  |  | % | 4.3% | 1.5% | 5.8% |  |
| HPV status  (oropharynx) | HPV16 (+) | N | 8 | 1 | 9 |  |
|  |  | % | 33.3% | 4.2% | 37.5% |  |
|  | HPV 58 (+) | N | 1 | 0 | 1 |  |
|  |  | % | 4.2% | 0.0% | 4.2% |  |
|  | HPV (-) | N | 4 | 1 | 5 |  |
|  |  | % | 16.6% | 4.2% | 20.8% |  |
|  | HPV N/A | N | 5 | 4 | 9 |  |
|  |  | % | 20.8% | 16.7% | 37.5% |  |
|  | p16 (+) | N | 9 | 1 | 10 |  |
|  |  | % | 37.5% | 4.2% | 41.7% |  |
|  | p16 (-) | N | 3 | 1 | 4 |  |
|  |  |  | 12.5% | 4.1 % | 16.6% |  |
|  | p16 N/A | N | 6 | 4 | 10 |  |
|  |  |  | 25% | 16.7% | 41.7% |  |
| Tumor stage  (AJCC) | I | N | 1 | 0 | 1 | 0.134 |
|  |  | % | 1.4% | 0.0% | 1.4% |  |
|  | II | N | 4 | 3 | 7 |  |
|  |  | % | 5.7% | 4.3% | 10% |  |
|  | III | N | 6 | 6 | 12 |  |
|  |  | % | 8.55% | 8.55% | 17.1% |  |
|  | IVA | N | 27 | 10 | 37 |  |
|  |  | % | 38.6% | 14.3% | 52.9% |  |
|  | IVB | N | 2 | 8 | 10 |  |
|  |  | % | 2.9% | 11.4% | 14.3% |  |
|  | IVC | N | 1 | 2 | 3 |  |
|  |  | % | 1.4% | 2.9% | 4.3% |  |
| Cisplatin-based chemoradiation | Adjuvant | N | 14 | 4 | 18 | 0.701 |
|  |  | % | 40.0% | 50.0% | 41.9% |  |
|  | Definitive | N | 21 | 4 | 25 |  |
|  |  | % | 60.0% | 50.0% | 58.1% |  |
| Systemic therapy | Cisplatin-  Olaparib | N | 1 | 2 | 3 |  |
|  |  | % | 3.7% | 7.4% | 11.1% |  |
|  | Olaparib | N | 1 | 2 | 3 |  |
|  |  | % | 3.7% | 7.4% | 11.1% |  |
|  | Durvalumab-Olaparib | N | 2 | 10 | 12 |  |
|  |  | % | 7.4% | 37.0% | 44.4% |  |
|  | Nivolumab |  | 2 | 7 | 9 |  |
|  |  |  | 7.4% | 26.0% | 33.4% |  |
|  | Total | N | 41 | 29 | 70 |  |
|  |  | % | 58.6% | 41.4% | 100.0% |  |

| **Supplementary Table S2. RT² Profiler™ PCR Array Human DNA Damage Signaling Pathway: Gene list** | | | |
| --- | --- | --- | --- |
| **Νο** | **Symbol** | **GeneBank** | **Description** |
| 1 | ABL1 | NM_005157 | C-abl oncogene 1, non-receptor tyrosine kinase |
| 2 | APEX1 | NM_080649 | APEX nuclease (multifunctional DNA repair enzyme) 1 |
| 3 | ATM | NM_000051 | Ataxia telangiectasia mutated |
| 4 | ATR | NM_001184 | Ataxia telangiectasia and Rad3 related |
| 5 | ATRIP | NM_032166 | ATR interacting protein |
| 6 | ATRX | NM_000489 | Alpha thalassemia/mental retardation syndrome X-linked |
| 7 | BARD1 | NM_000465 | BRCA1 associated RING domain 1 |
| 8 | BAX | NM_004324 | BCL2-associated X protein |
| 9 | BBC3 | NM_014417 | BCL2 binding component 3 |
| 10 | BLM | NM_000057 | Bloom syndrome, RecQ helicase-like |
| 11 | BRCA1 | NM_007294 | Breast cancer 1, early onset |
| 12 | BRIP1 | NM_032043 | BRCA1 interacting protein C-terminal helicase 1 |
| 13 | CDC25A | NM_001789 | Cell division cycle 25 homolog A (S. pombe) |
| 14 | CDC25C | NM_001790 | Cell division cycle 25 homolog C (S. pombe) |
| 15 | CDK7 | NM_001799 | Cyclin-dependent kinase 7 |
| 16 | CDKN1A | NM_000389 | Cyclin-dependent kinase inhibitor 1A (p21, Cip1) |
| 17 | CHEK1 | NM_001274 | CHK1 checkpoint homolog (S. pombe) |
| 18 | CHEK2 | NM_007194 | CHK2 checkpoint homolog (S. pombe) |
| 19 | CIB1 | NM_006384 | Calcium and integrin binding 1 (calmyrin) |
| 20 | CRY1 | NM_004075 | Cryptochrome 1 (photolyase-like) |
| 21 | CSNK2A2 | NM_001896 | Casein kinase 2, alpha prime polypeptide |
| 22 | DDB1 | NM_001923 | Damage-specific DNA binding protein 1, 127kDa |
| 23 | DDB2 | NM_000107 | Damage-specific DNA binding protein 2, 48kDa |
| 24 | DDIT3 | NM_004083 | DNA-damage-inducible transcript 3 |
| 25 | ERCC1 | NM_001983 | Excision repair cross-complementing rodent repair deficiency, complementation group 1 (includes overlapping antisense sequence) |
| 26 | ERCC2 | NM_000400 | Excision repair cross-complementing rodent repair deficiency, complementation group 2 |
| 27 | EXO1 | NM_130398 | Exonuclease 1 |
| 28 | FANCA | NM_000135 | Fanconi anemia, complementation group A |
| 29 | FANCD2 | NM_033084 | Fanconi anemia, complementation group D2 |
| 30 | FANCG | NM_004629 | Fanconi anemia, complementation group G |
| 31 | FEN1 | NM_004111 | Flap structure-specific endonuclease 1 |
| 32 | GADD45A | NM_001924 | Growth arrest and DNA-damage-inducible, alpha |
| 33 | GADD45G | NM_006705 | Growth arrest and DNA-damage-inducible, gamma |
| 34 | H2AFX | NM_002105 | H2A histone family, member X |
| 35 | HUS1 | NM_004507 | HUS1 checkpoint homolog (S. pombe) |
| 36 | LIG1 | NM_000234 | Ligase I, DNA, ATP-dependent |
| 37 | MAPK12 | NM_002969 | Mitogen-activated protein kinase 12 |
| 38 | MBD4 | NM_003925 | Methyl-CpG binding domain protein 4 |
| 39 | MCPH1 | NM_024596 | Microcephalin 1 |
| 40 | MDC1 | NM_014641 | Mediator of DNA-damage checkpoint 1 |
| 41 | MLH1 | NM_000249 | MutL homolog 1, colon cancer, nonpolyposis type 2 (E. coli) |
| 42 | MLH3 | NM_014381 | MutL homolog 3 (E. coli) |
| 43 | MPG | NM_002434 | N-methylpurine-DNA glycosylase |
| 44 | MRE11A | NM_005590 | MRE11 meiotic recombination 11 homolog A (S. cerevisiae) |
| 45 | MSH2 | NM_000251 | MutS homolog 2, colon cancer, nonpolyposis type 1 (E. coli) |
| 46 | MSH3 | NM_002439 | MutS homolog 3 (E. coli) |
| 47 | NBN | NM_002485 | Nibrin |
| 48 | NTHL1 | NM_002528 | Nth endonuclease III-like 1 (E. coli) |
| 49 | OGG1 | NM_002542 | 8-oxoguanine DNA glycosylase |
| 50 | PARP1 | NM_001618 | Poly (ADP-ribose) polymerase 1 |
| 51 | PCNA | NM_182649 | Proliferating cell nuclear antigen |
| 52 | PMS1 | NM_000534 | PMS1 postmeiotic segregation increased 1 (S. cerevisiae) |
| 53 | PMS2 | NM_000535 | PMS2 postmeiotic segregation increased 2 (S. cerevisiae) |
| 54 | PNKP | NM_007254 | Polynucleotide kinase 3'-phosphatase |
| 55 | PPM1D | NM_003620 | Protein phosphatase, Mg2+/Mn2+ dependent, 1D |
| 56 | PPP1R15A | NM_014330 | Protein phosphatase 1, regulatory (inhibitor) subunit 15A |
| 57 | PRKDC | NM_006904 | Protein kinase, DNA-activated, catalytic polypeptide |
| 58 | RAD1 | NM_002853 | RAD1 homolog (S. pombe) |
| 59 | RAD17 | NM_002873 | RAD17 homolog (S. pombe) |
| 60 | RAD18 | NM_020165 | RAD18 homolog (S. cerevisiae) |
| 61 | RAD21 | NM_006265 | RAD21 homolog (S. pombe) |
| 62 | RAD50 | NM_005732 | RAD50 homolog (S. cerevisiae) |
| 63 | RAD51 | NM_002875 | RAD51 homolog (S. cerevisiae) |
| 64 | RAD51B | NM_133509 | RAD51 homolog B (S. cerevisiae) |
| 65 | RAD9A | NM_004584 | RAD9 homolog A (S. pombe) |
| 66 | RBBP8 | NM_002894 | Retinoblastoma binding protein 8 |
| 67 | REV1 | NM_016316 | REV1 homolog (S. cerevisiae) |
| 68 | RNF168 | NM_152617 | Ring finger protein 168 |
| 69 | RNF8 | NM_183078 | Ring finger protein 8 |
| 70 | RPA1 | NM_002945 | Replication protein A1, 70kDa |
| 71 | SIRT1 | NM_012238 | Sirtuin 1 |
| 72 | SMC1A | NM_006306 | Structural maintenance of chromosomes 1A |
| 73 | SUMO1 | NM_003352 | SMT3 suppressor of mif two 3 homolog 1 (S. cerevisiae) |
| 74 | TOPBP1 | NM_007027 | Topoisomerase (DNA) II binding protein 1 |
| 75 | TP53 | NM_000546 | Tumor protein p53 |
| 76 | TP53BP1 | NM_005657 | Tumor protein p53 binding protein 1 |
| 77 | TP73 | NM_005427 | Tumor protein p73 |
| 78 | UNG | NM_003362 | Uracil-DNA glycosylase |
| 79 | XPA | NM_000380 | Xeroderma pigmentosum, complementation group A |
| 80 | XPC | NM_004628 | Xeroderma pigmentosum, complementation group C |
| 81 | XRCC1 | NM_006297 | X-ray repair complementing defective repair in Chinese hamster cells 1 |
| 82 | XRCC2 | NM_005431 | X-ray repair complementing defective repair in Chinese hamster cells 2 |
| 83 | XRCC3 | NM_005432 | X-ray repair complementing defective repair in Chinese hamster cells 3 |
| 84 | XRCC6 | NM_001469 | X-ray repair complementing defective repair in Chinese hamster cells 6 |

| **Supplementary Table S3. Correlations between individual DDR parameters at baseline and patients’ characteristics using** **Mann-Whitney U test** | | | | |
| --- | --- | --- | --- | --- |
| **Patients’ characteristic** | **Endogenous DNA damage (Olive tail moment)** | **DSB repair capacity at baseline** | **Oxidative stress**  **at baseline** | **Abasic sites**  **at baseline** |
|  | ***P*-value** | | | |
| Age | 0.779 | 0.160 | 0.472 | 0.386 |
| Gender | 0.229 | 0.684 | 0.373 | 0.080 |
| Alcohol excess | 0.716 | 0.666 | 0.666 | 0.702 |
| Smoking history | 0.217 | 0.105 | 0.109 | 0.222 |

| **Supplementary Table S4. Cox proportional hazard model demonstrating the independent prognostic significance for progression-free survival of NER capacity (expressed as DNA damage AUC)** | | | | | |
| --- | --- | --- | --- | --- | --- |
| **Covariates** | **Values** | **HR** | **95% CI of HR** | | ***P*-value** |
| Age (years) | ≥60 *vs*. < 60 | 1.016 | 0.287 | 3.594 | 0.981 |
| Gender | Male *vs*. female | 0.317 | 0.074 | 1.359 | 0.122 |
| Primary site | Larynx *vs*. other | 1.579 | 0.340 | 7.335 | 0.560 |
| Stage | IV *vs*. I-III | 0.726 | 0.188 | 2.805 | 0.642 |
| DNA damage AUC | Increasing | 0.998 | 0.996 | 1.000 | 0.037 |
| AUC, area under the curve; CI, confidence intervals; HR, hazard ratio | | | | | |

| **Supplementary Table S5. Cox proportional hazard model demonstrating the independent prognostic significance for progression-free survival of apoptosis (expressed as cisplatin dose inducing apoptosis)** | | | | | |
| --- | --- | --- | --- | --- | --- |
| **Covariates** | **Values** | **HR** | **95% CI of HR** | | ***P*-value** |
| Age (years) | ≥60 *vs*. < 60 | 1.443 | 0.412 | 5.055 | 0.567 |
| Gender | Male *vs*. female | 0.298 | 0.068 | 1.309 | 0.109 |
| Primary site | Larynx *vs*. other | 2.087 | 0.431 | 10.106 | 0.360 |
| Stage | IV *vs*. I-III | 1.446 | 0.344 | 6.076 | 0.614 |
| Cisplatin dose inducing apoptosis | Increasing | 1.060 | 1.006 | 1.118 | 0.029 |
| CI, confidence intervals; HR, hazard ratio | | | | | |

**
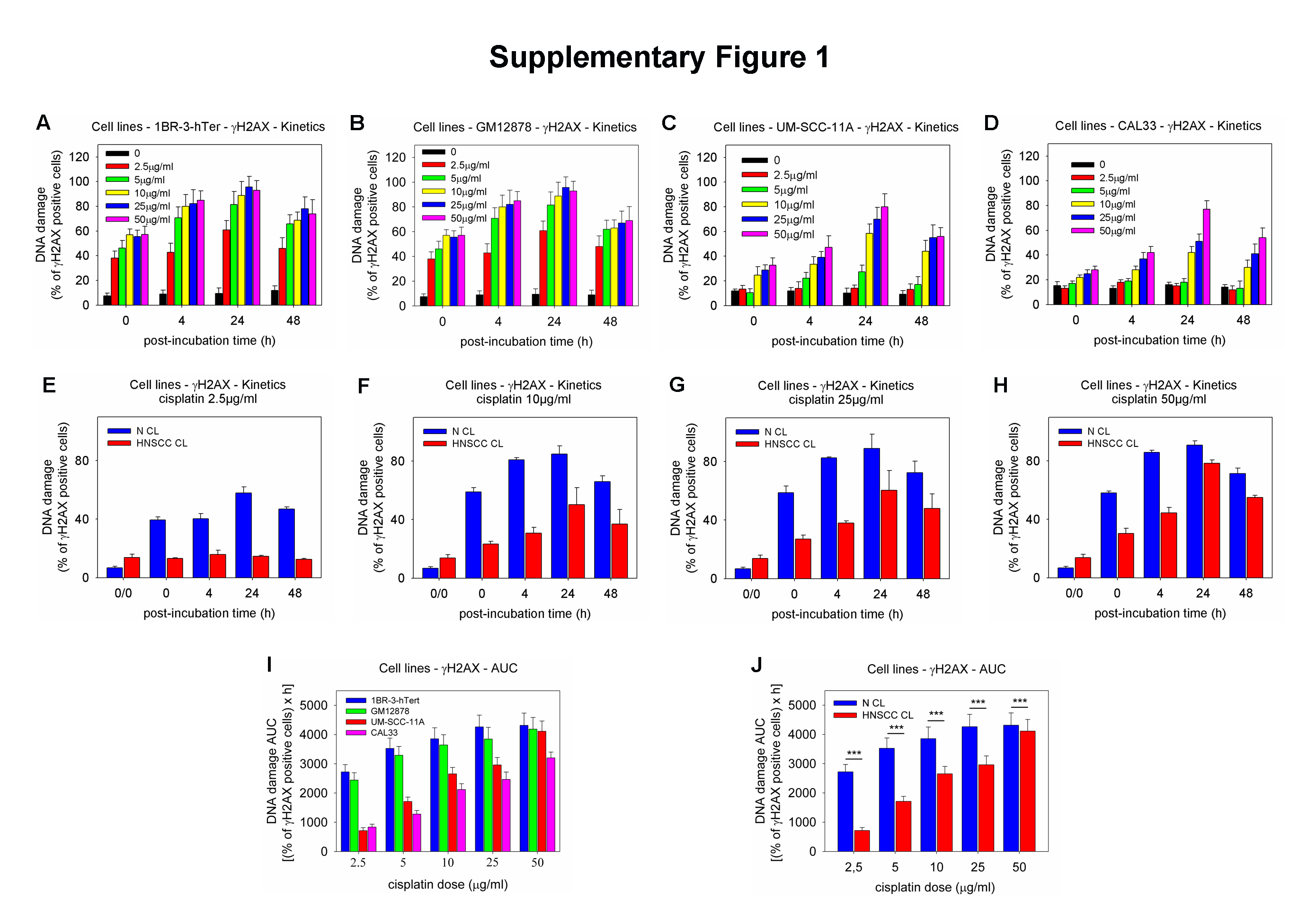
**

**Supplementary Figure S1. DSB formation and repair using immunofluorescence γH2AX staining and confocal microscopy in cell lines.** (A-H) Bar charts showing the kinetics of γH2AX foci formation/removal, and (I, J) the accumulation of γH2AX foci, expressed as AUC, following treatment of normal (1BR-3-hTert, GM12878) and HNSCC (UM-SCC-11A, CAL33) cell lines with various doses of cisplatin (0-50μg/ml). N CL, normal cell line; HNSCC CL, HNSCC cell line. All error bars represent SD; ****P* < 0.001 by Mann-Whitney U test. The experiments shown were based on a minimum of 3 independent repeats.


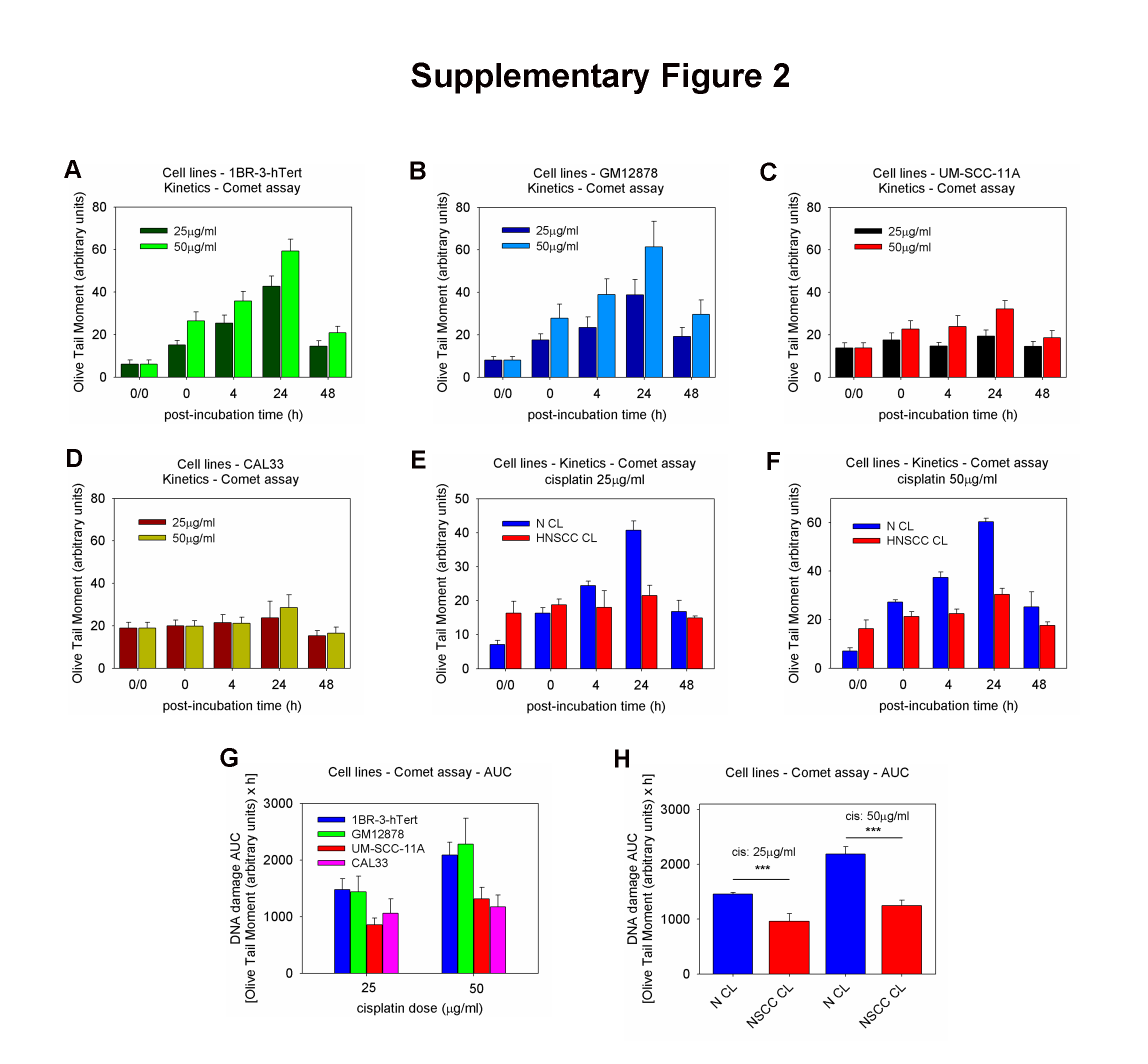


**Supplementary Figure S2. DSB formation and repair using neutral comet assay in cell lines**. Bar charts showing (A-F) the kinetics of DSB formation/repair, and (G, H) the accumulation of DSBs, expressed as AUC for DNA damage during the whole experiment (0-48h). All error bars represent SD; ****P* < 0.001 by Mann-Whitney U test. The experiments shown were based on a minimum of 3 independent repeats.


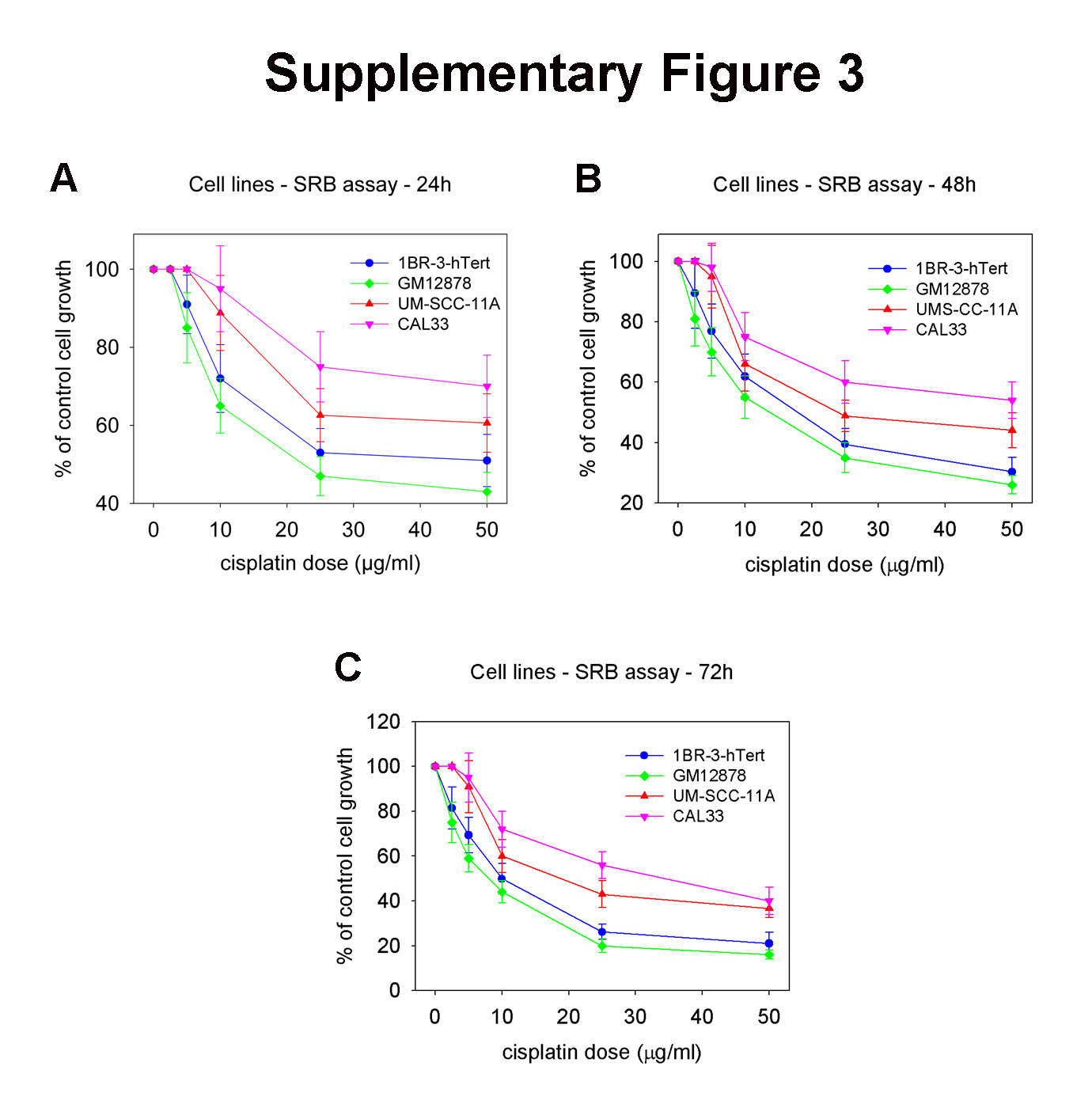


**Supplementary Figure S3. Cytotoxicity and cell proliferation in cell lines**. Drug-induced cytotoxicity and cell proliferation using the SRB assay at 24h (A), 48h (B) and 72h (C) after cisplatin treatment. The experiments shown were based on a minimum of 3 independent repeats.


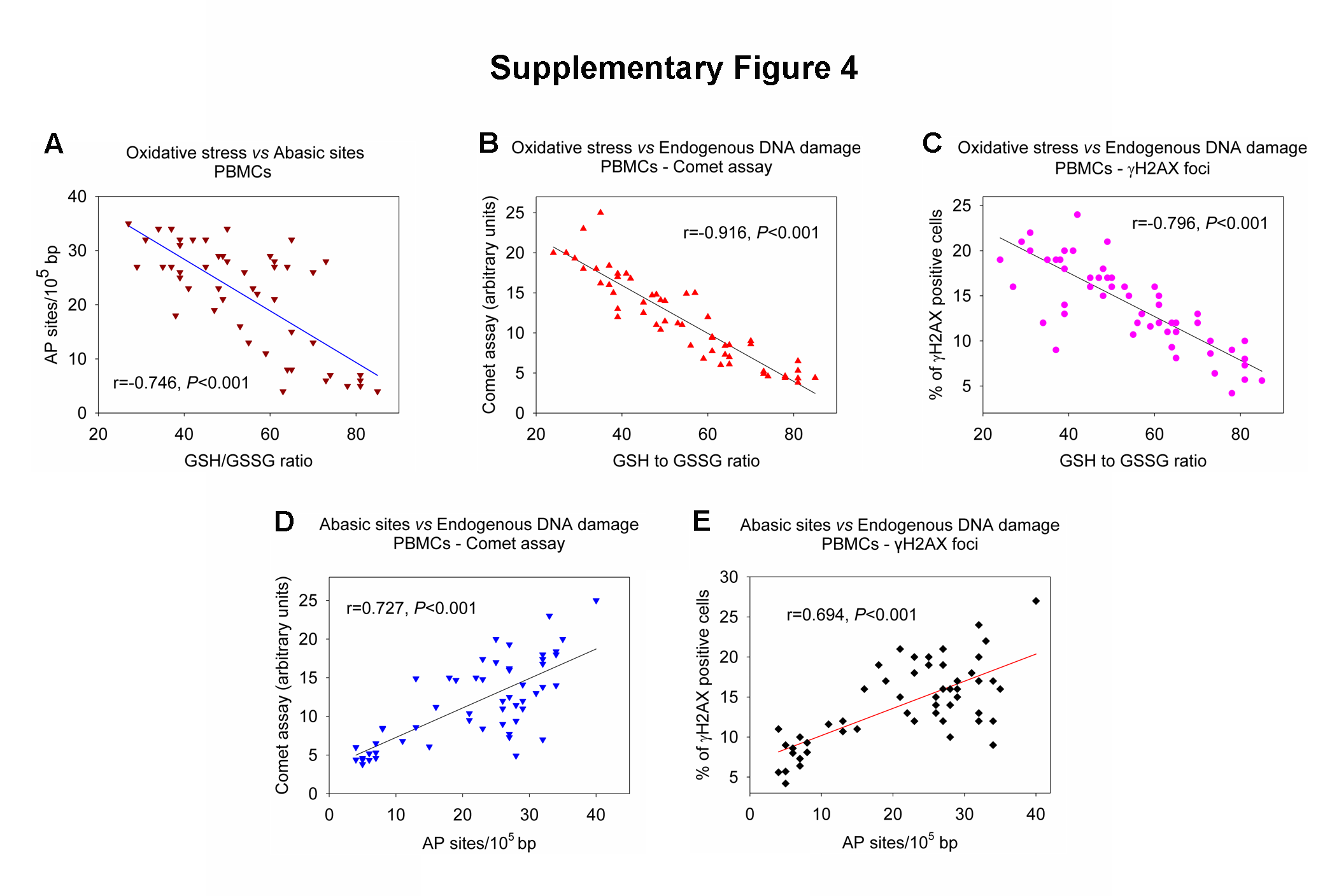


**Supplementary Figure S4. Correlations between the endogenous DNA damage, oxidative stress and abasic sites in PBMCs at baseline**. (A) Correlation between oxidative stress and abasic sites in PBMCs from the same patients. Correlations between oxidative stress and the individual endogenous DNA damage measured (B) by the alkaline comet assay or (C) the immunofluorescence γH2AX staining. Correlations between AP-sites and the individual endogenous DNA damage measured by (D) the alkaline comet assay or (E) the immunofluorescence γH2AX staining are also presented. The experiments shown were based on a minimum of 3 independent repeats.


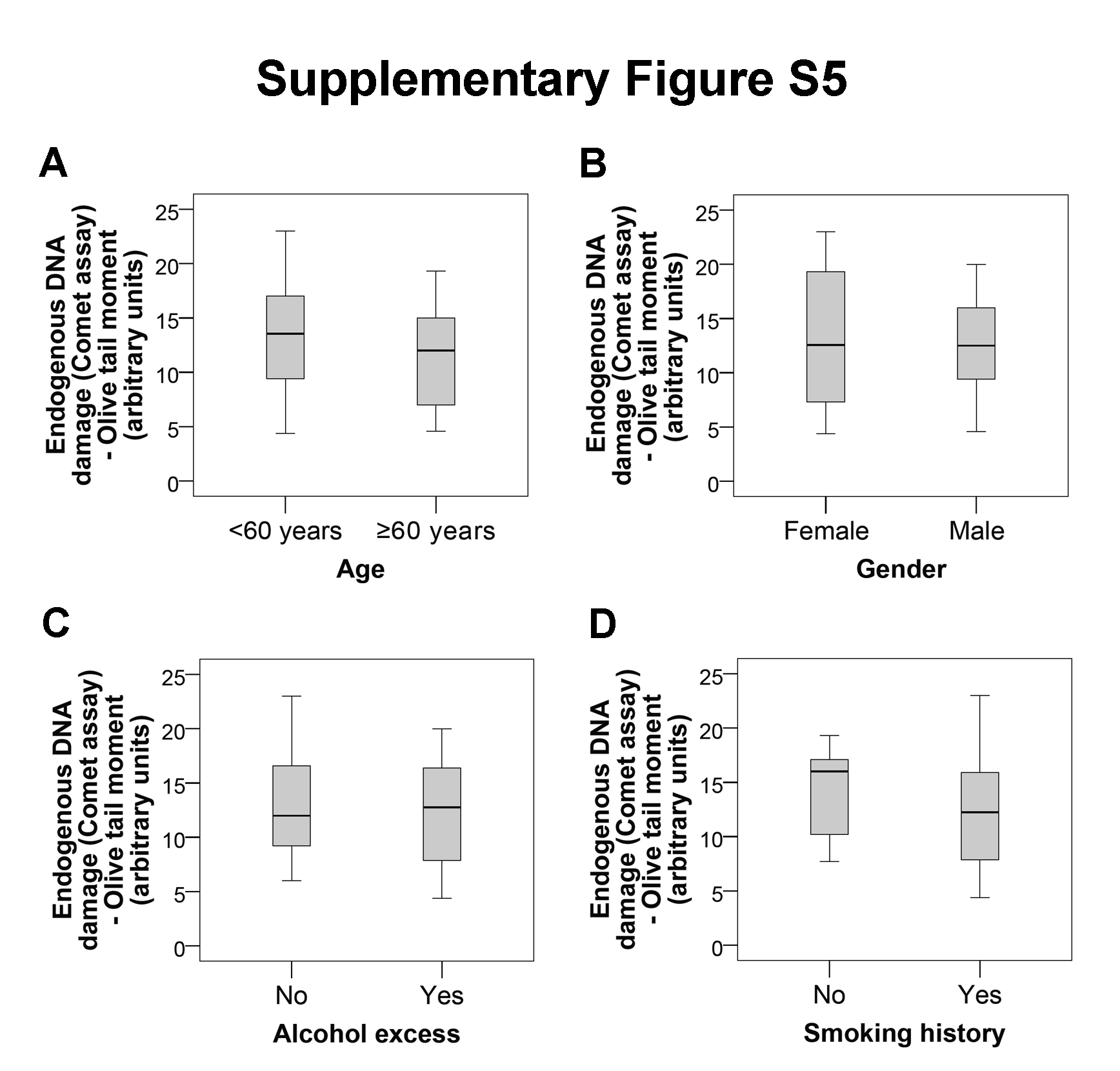


**Supplementary Figure S5. Correlations between endogenous DNA damage and patients’ characteristics using** **Mann-Whitney U test.** Box plots for the distribution of the endogenous DNA damage divided according to (A) age, (B) gender, (C) alcohol excess, and (D) smoking history.


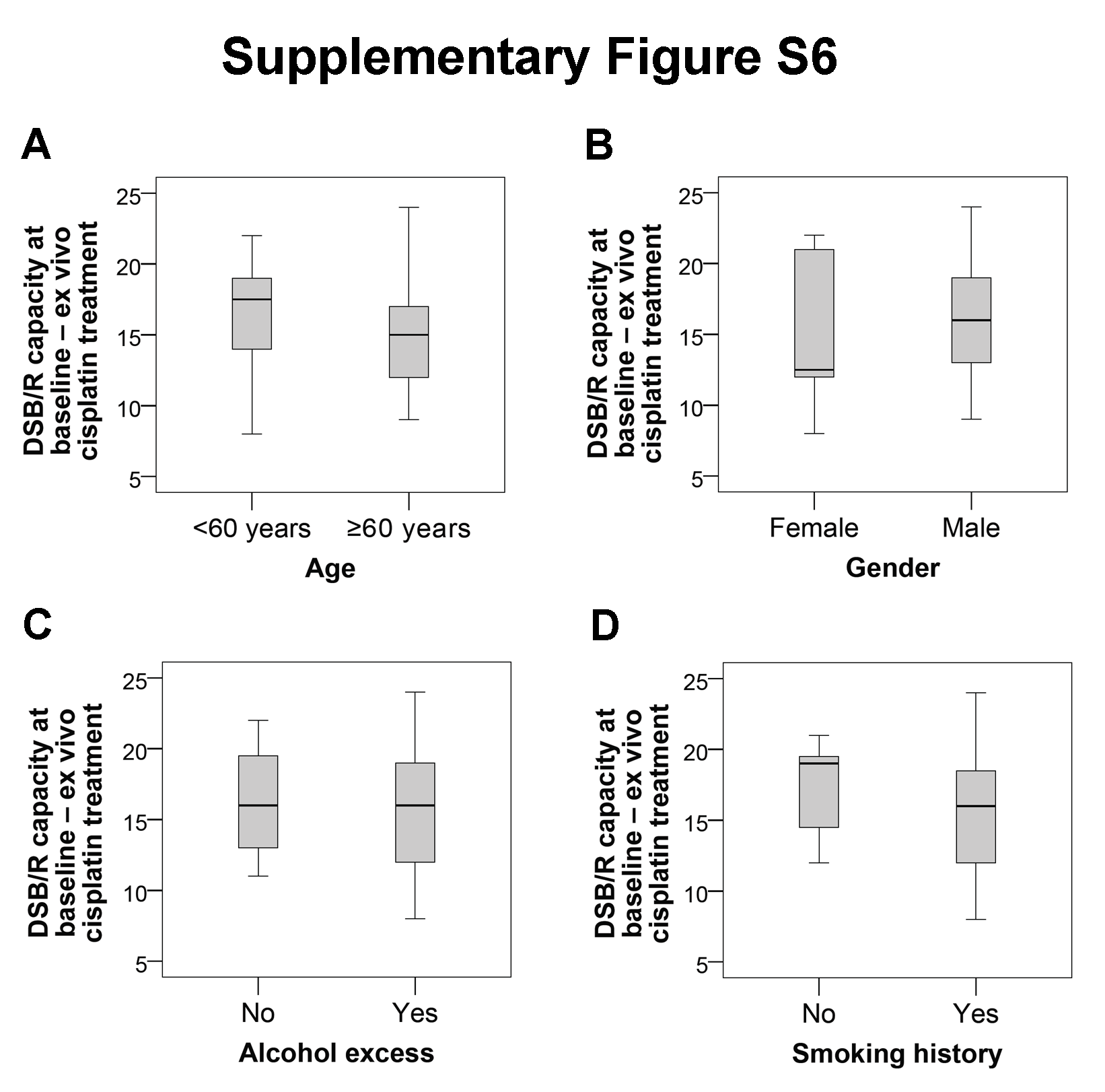


**Supplementary Figure S6. Correlations between DSB repair capacity at baseline and patients’ characteristics using** **Mann-Whitney U test.** Box plots for the distribution of the DSB repair (DSB/R) capacity at baseline divided according to (A) age, (B) gender, (C) alcohol excess, and (D) smoking history.


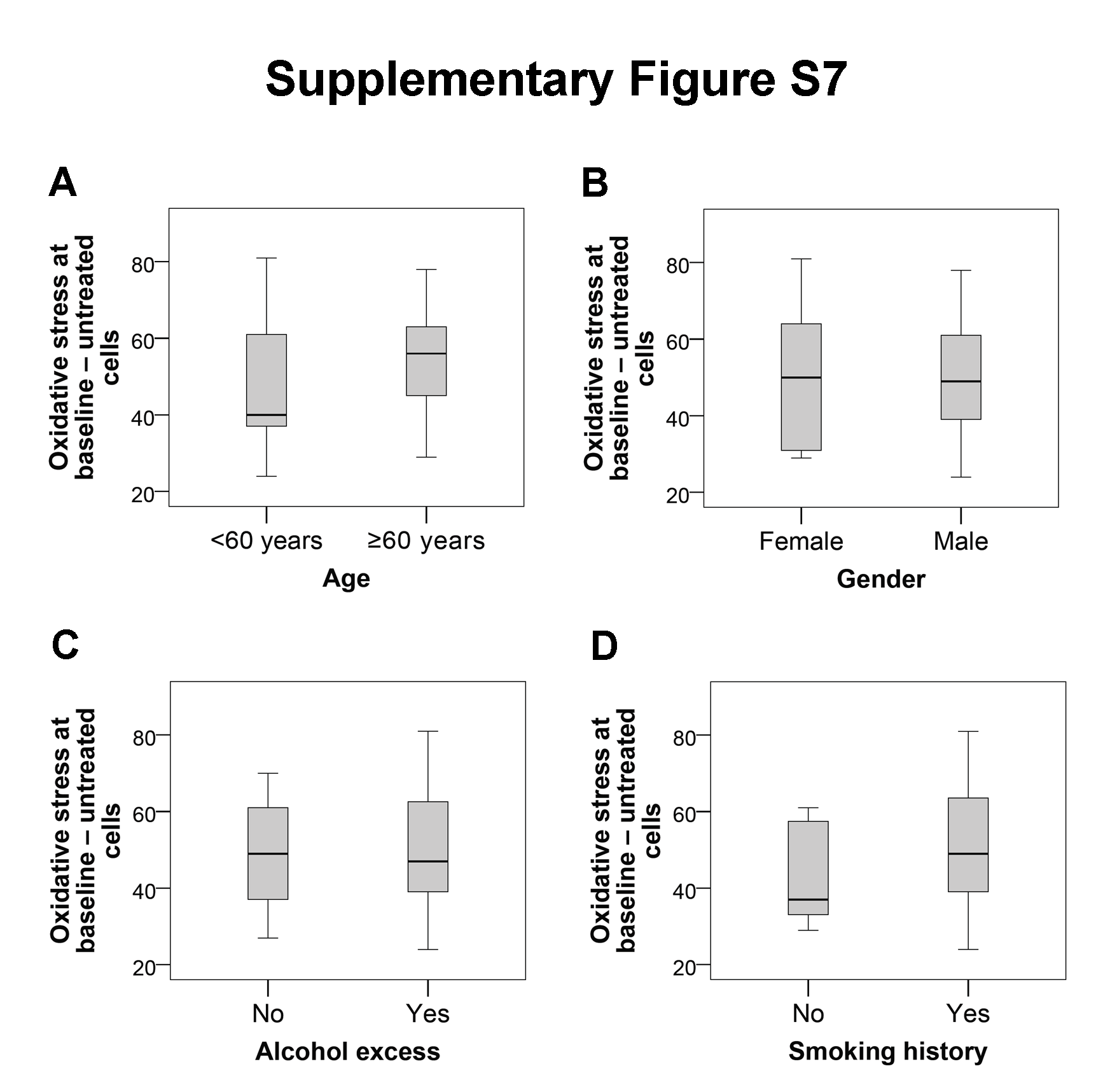


**Supplementary Figure S7. Correlations between oxidative stress at baseline and patients’ characteristics using** **Mann-Whitney U test.** Box plots for the distribution of the oxidative stress at baseline divided according to (A) age, (B) gender, (C) alcohol excess, and (D) smoking history.


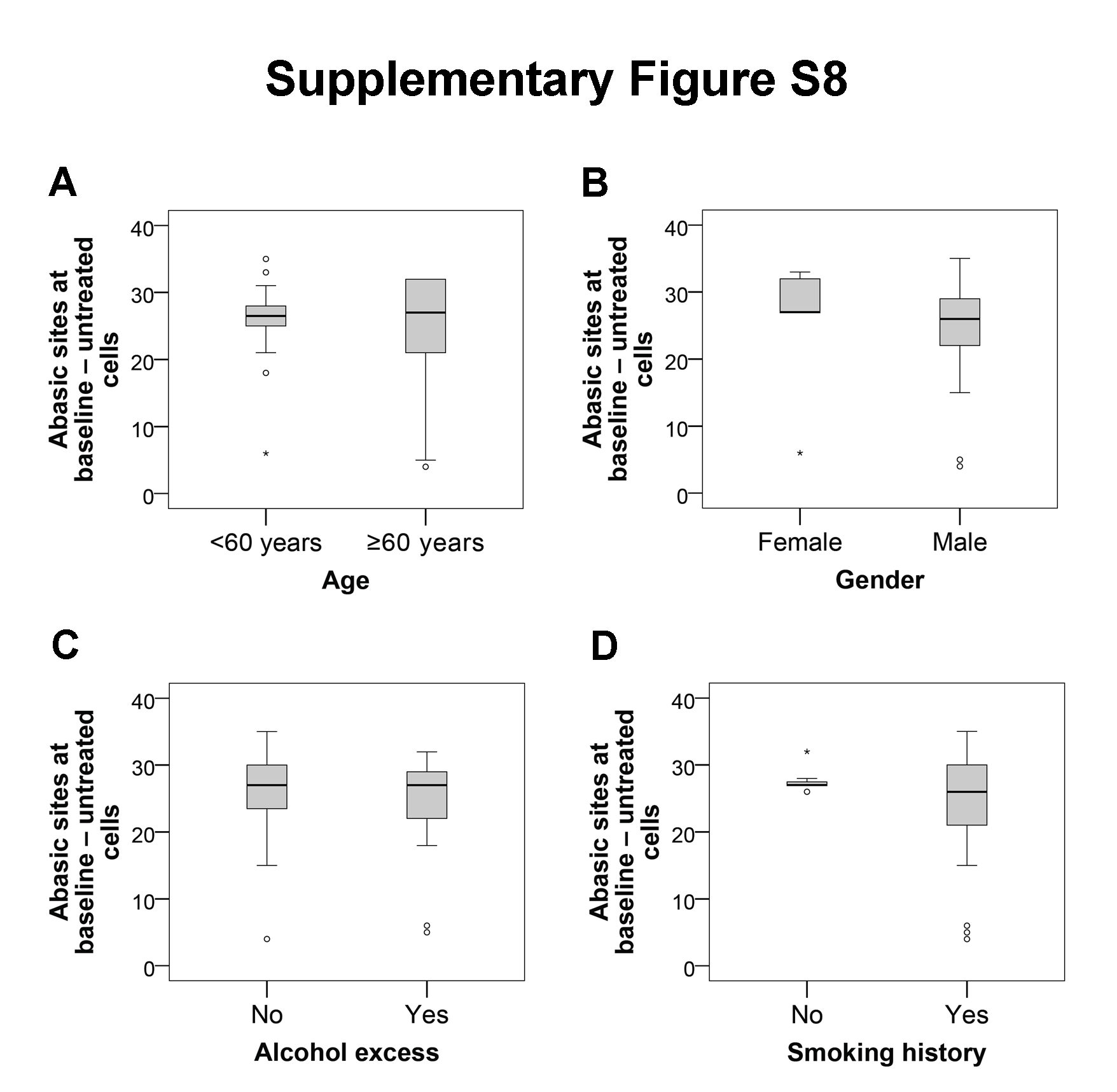


**Supplementary Figure S8. Correlations between abasic sites at baseline and patients’ characteristics using** **Mann-Whitney U test.** Box plots for the distribution of the abasic sites at baseline divided according to (A) age, (B) gender, (C) alcohol excess, and (D) smoking history.
